# Treating energy‐based devices and hyaluronic acid filler injection together?

**DOI:** 10.1111/srt.13716

**Published:** 2024-04-17

**Authors:** Kyu‐Ho Yi

**Affiliations:** ^1^ Division in Anatomy and Developmental Biology Department of Oral Biology Human Identification Research Institute BK21 FOUR Project Yonsei University College of Dentistry Seoul South Korea; ^2^ Maylin Clinic (Apgujeong) Seoul South Korea

Dear Editor,

Dermal fillers, particularly hyaluronic acid (HA) fillers, are widely used for facial volume enhancement and shaping. HA is a polysaccharide naturally found in the dermis, known for its ability to be synthesized and broken down within human tissues. However, factors such as enzymes, pH levels, ultrasound, temperature changes, oxidative stress, and UV exposure can speed up HA degradation. Various technologies like cross‐linking and additive incorporation have been developed to stabilize HA.^1^, ^2^, ^3^, ^4^, ^5^

Energy‐based devices (EBD) like Radiofrequency (RF), Multi‐wavelength diode laser (MWDL), and High‐intensity Focused Ultrasound (HIFU) are used for skin tightening, fat reduction, and collagen stimulation. HIFU generates rapid heating, causing protein denaturation and cell death. MWDL targets deeper skin layers and can stimulate interest in combined therapy with fillers. These devices generally have minimal downtime and are preferred for skin tightening.

To maintain the integrity of HA fillers, it's recommended to use EBD before HA filler injection to reduce the risk of filler degradation. However, caution is advised when using EBD that penetrates deeply into tissues after HA injection as it may compromise the filler's integrity and effectiveness.

Studies have shown that HA solutions display retrograde behavior with decreased viscosity at elevated temperatures. High temperatures can cause significant HA degradation. Current cross‐linking methods often involve autoclaving at about 120°C, highlighting the need for further investigation into the effects of temperature changes, such as the approximately 70°C change induced by EBD.^6^, ^7^, ^8^

HA fillers are primarily temporary and efforts to extend their lifespan have led to innovations in crosslinking technology. However, various factors can accelerate HA breakdown, including enzymatic activity, pH changes, ultrasound exposure, high temperatures, oxidative stress, and UV light.

EBD are increasingly popular for cosmetic procedures due to their ability to generate heat and stimulate collagen production. The ideal temperature for EBD varies depending on the treatment goals, but excessive heat can accelerate HA degradation. Therefore, it's generally recommended to separate EBD and HA filler treatments, although the optimal time interval between them remains unclear.

Studies have produced conflicting results on the impact of EBD heat on intradermal HA fillers. Some studies suggest that early EBD treatment may result in HA filler degradation, while others indicate that delaying EBD treatment can reduce this effect. Additionally, certain conditions like bleeding in the subcutaneous layer or the presence of specific fillers can increase the risk of burn injuries during EBD treatments.^8^

In conclusion, the interaction between EBD and HA fillers varies depending on factors like the type of EBD, energy level, type of HA filler, injection depth, and time interval between treatments. Future studies should include a larger participant pool, a wider range of products, and different EBD technologies to provide comprehensive insights and guidelines for optimal time intervals between HA filler injection and EBD treatment. Other fillers like calcium hydroxyapatite, polycaprolactone, and polynucleotides should also be analyzed to understand their interactions with EBD better.

## CONFLICT OF INTEREST STATEMENT

There is no conlict of interest.

## Data Availability

The data that support the findings of this study are available from the corresponding author upon reasonable request.
